# Dental and Periodontal Health in Acute Intermittent Porphyria

**DOI:** 10.3390/life12081270

**Published:** 2022-08-19

**Authors:** Elin Storjord, Stella Airila-Månsson, Katarzyna Karlsen, Martin Madsen, Jim André Dahl, Anne Landsem, Hilde Fure, Judith Krey Ludviksen, Johannes Østrem Fjøse, Amy K. Dickey, Bård Ove Karlsen, Erik Waage Nielsen, Tom Eirik Mollnes, Ole-Lars Brekke

**Affiliations:** 1 Department of Laboratory Medicine, Nordland Hospital Trust, 8092 Bodø, Norway; 2 Department of Clinical Medicine, UiT—The Arctic University of Norway Tromsø, 9019 Tromsø, Norway; 3 Public Dental Health District Midt, 8092 Bodø, Norway; 4 Public Dental Health Care Service, 8250 Rognan, Norway; 5 Department of Oral, Maxillofacial and Dental Surgery, Nordland Hospital Trust, 8092 Bodø, Norway; 6 Research Laboratory, Nordland Hospital Trust, 8092 Bodø, Norway; 7 Fürst Medical Laboratory, 0450 Oslo, Norway; 8 Department of Medicine, Massachusetts General Hospital, Boston, MA 02114, USA; 9 Harvard Medical School, Boston, MA 02115, USA; 10 Faculty of Nursing and Health Sciences, Nord University, 8092 Bodø, Norway; 11 Department of Anesthesiology, Nordland Hospital Trust, 8092 Bodø, Norway; 12 Department of Immunology, University of Oslo and Oslo University Hospital, 0450 Oslo, Norway; 13 Centre of Molecular Inflammation Research, Norwegian University of Science and Technology, 7034 Trondheim, Norway

**Keywords:** cytokines, inflammation, immunity, innate immunity, periodontitis, periodontal disease, systemic health, delta aminolevulinic acid, porphobilinogen

## Abstract

In the inherited metabolic disorder acute intermittent porphyria (AIP), high sugar intake prevents porphyric attacks due to the glucose effect and the following high insulin levels that may lower AIP disease activity. Insulin resistance is a known risk factor for periodontitis and sugar changes diabetogenic hormones and affects dental health. We hypothesized differences in homeostasis model assessment (HOMA) scores for insulin resistance in AIP cases vs. controls and in those with periodontitis. Our aim was to systematically study dental health in AIP as poor dental health was previously only described in case reports. Further, we aimed to examine if poor dental health and kidney failure might worsen AIP as chronic inflammation and kidney failure might increase disease activity. In 47 AIP cases and 47 matched controls, X-rays and physical examination of clinical attachment loss (CAL), probing pocket depth (PPD), and decayed missing filled teeth (DMFT) were performed. Dietary intake was evaluated through a diet logbook. Plasma cytokines and diabetogenic hormones were measured using multiplex technology and urine porphobilinogen and kidney and liver function by routine methods. An excel spreadsheet from the University of Oxford was used to estimate HOMA scores; beta cell function, HOMA%B (%B), insulin sensitivity, HOMA%S (%S), and insulin resistance HOMA-IR (IR), based on glucose and plasma (P) C-peptide. The Wilcoxon matched-pairs signed rank test, the Mann–Whitney U-test, and Spearman’s non-parametric correlation were used. Insulin (*p* = 0.007) and C-peptide (*p* = 0.006) were higher in the AIP cases with periodontitis versus those without. In AIP patients, the liver fibrosis index 4 correlated with DMFT (*p* < 0.001) and CAL ≥4 mm (*p* = 0.006); the estimated glomerular filtration rate correlated with DMFT (*p* < 0.001) and CAL ≥4 mm (*p* = 0.02). CAL ≥4 mm was correlated with chemokine ligand 11 and interleukin (IL)-13 *(p* = 0.04 for both), and PPD >5 mm was correlated with plasminogen activator inhibitor-1 (*p* = 0.003) and complement component 3 (*p* = 0.02). In conclusion, dental health in AIP cases was correlated with insulin resistance, inflammatory markers, and biomarkers of kidney and liver function, demonstrating that organ damage in the kidney and liver are associated with poorer dental health.

## 1. Introduction

Acute intermittent porphyria (AIP) is an inherited metabolic disease caused by a mutation in the hydroxymethylbilane synthase (HMBS) gene leading to reduced enzymatic function [1,2,3,4]. Diagnosis relies on genetic tests, urine porphobilinogen (U-PBG), and delta-aminolevulinic acid (U-ALA). AIP patients might have acute attacks of abdominal pain, nausea, vomiting, and fatigue that can be triggered by a low carbohydrate diet, drugs, stress, hormonal changes, infections, and alcohol [5,6]. Furthermore, renal failure and hepatocellular carcinoma are also complications of the disease [5,7].

Insufficient carbohydrate intake might induce AIP attacks and a carbohydrate intake of 55–60% of total energy intake is recommended. Carbohydrate loading is especially important when patients begin to have symptoms of an emerging attack [5]. Glucose acts by repressing heme synthesis in the liver by downregulating the 5-aminolevulinate synthase 1 (ALAS-1) enzyme production and thereby decreasing the accumulation of toxic biochemical intermediates. In the nucleus of hepatocytes, the peroxisome proliferator activated cofactor-1 alpha (PGC-1α) binds with forkhead box protein O1 (FOXO1) and increases ALAS-1 gene expression. The introduction of glucose leads to the production of insulin that, via receptors on the cell membrane, activates phosphoinositide 3-kinases (PI3K) and consequentially protein kinase B (AKT). This in turn phosphorylates FOXO1. The phosphorylated FOXO1 is carried out of the nucleus, and the synergistic activation with PGC-1α on the ALAS-1 gene is inhibited [8,9,10]. A high sugar intake prevents porphyric attacks but could change diabetogenic hormones and affect dental health. Particularly, periodontitis sometimes goes unrecognized by the patients, and chronic inflammation from periodontitis might increase AIP disease activity [11,12,13].

Complement inhibition is proposed for the treatment of periodontitis [14,15]. Periodontitis itself is linked to high blood sugar, inflammation, smoking, diabetes, and chronic kidney disease [16]. Similarly, AIP patients are known to have low-grade systemic inflammation, and symptomatic AIP cases additionally have decreased C-peptide, insulin release, and kidney function [11]. Complications of AIP such as kidney and liver disease, chronic inflammation, vomiting during attacks, and a high attack frequency could potentially worsen dental health. We also hypothesized differences in homeostasis model assessment (HOMA) scores for insulin resistance in AIP cases vs. controls and in those with periodontitis.

Only a few case reports describe AIP and dental health. These reports focus on whether different types of local analgesic drugs, general anaesthesia, and antibiotics are porphyrogenic and on this basis which ones should be recommended or avoided as part of dental treatment [17,18]. McGovern et al. showed the uneventful use of bupivacaine in five persons with latent AIP [18]. Brown et al. reported the management of a dental abscess in AIP, and states that a maintenance of a good chewing function is especially important in AIP since a too-low energy intake is a trigger for AIP attacks. Infection is also a trigger of AIP attacks and the treatment of infections is therefore also very important in AIP [17]. Witbeck suggested that a severe dental infection in AIP might have been linked to the recommended high intake of carbohydrates [19]. A review of Moore et al. on acute porphyric disorders lists seven recommendations for the dental management of patients with acute porphyric disorders, one being avoid the attack trigger barbiturates [20]. Next, the dental practitioner should ask about the persons diet and provide appropriate oral hygiene instructions and follow up-visits every 3 to 6 months due to the recommended high carbohydrate intake [20].

Our aim was to systematically study dental health in AIP. Further, our aim was to examine if poor dental health and kidney failure might worsen AIP as chronic inflammation and kidney failure might increase disease activity. We therefore examined dental health, diet, liver and kidney function, inflammation, diabetogenic hormones, and AIP biochemical disease activity in AIP cases and matched healthy controls.

## 2. Materials and Methods

### 2.1. Participants and Study Design

A case-control study of 47 adults with AIP and 47 controls, matched for age, sex, and residence was conducted from September to November 2012 in Bodø, Nordland County, Norway. The persons with AIP and the matched controls were recruited from the Norwegian counties of Nordland, Troms, Trøndelag, and Oslo. Of the AIP cases, 15 were asymptomatic and 32 symptomatic. Of the initial 50 AIP cases and 50 controls, three AIP cases and one control were edentulous. These and their matched controls were excluded [11]. Another two AIP cases and their corresponding controls were excluded only from CAL measurements because the two AIP cases had crowns covering their natural teeth, which made it impossible to measure CAL.

We had no data, prior to the study, regarding the expected mean difference on dental health between the healthy control group and a group of persons with the rare disease AIP. We anticipated a difference in PBG in urine of 9 μmol/mmol creatinine between the mean value of the healthy controls and the AIP cases [21]. In that regard, we needed a minimum of 20 persons to find a statistical difference on PBG between the groups with 95% probability (*p* < 0.05) at the previously observed SD for the mean value. Reviewing literature regarding dental research, we found that sample size often was larger than this in order to find statistical difference. As a practically possible approach for full dental examination of the rare disease AIP, we included 50 AIP cases and 50 healthy controls.

### 2.2. Ethical Considerations

All the procedures in this trial were in accordance with the ethical standards of the responsible committee on human experimentation (institutional and national) and with the Helsinki Declaration. The Regional Committee for Medical and Health Research Ethics approved the study: approval number 2011/2197. ClinicalTrials.gov Identifier: NCT01617642. Written informed consent was obtained from all participants, and the study conforms to STROBE Guidelines (Table S1) [22].

### 2.3. Examination of Dental Health

One dentist evaluated all the bitewing X-rays in conjunction with the clinical examination. The result from this examination in addition to the bitewing X-ray was given as the number of decayed teeth, missing teeth, and filled teeth (DMFT). To minimize differences in examination technique, the dentist had, prior to the study, tested the research examination procedure. The procedure was done on individuals not part of the study, to approximate time use and to rehearse a standardized procedure.

Orthopantograms (OPG) and bitewing X-rays were performed. One oral surgeon evaluated all the orthopantograms thoroughly by visual examination of the OPG on screen. The results were given as missing teeth from OPG, apical periodontitis from OPG, and wisdom tooth present from OPG. Further, the number of decayed teeth, filled teeth, root canal fillings, abnormal tooth positions, crowns, bridge pillars, inflamed root canal fillings, root rests, and implants were found on the OPG.

Periodontitis was assessed by probe UNC15 with defined spring force 0.2 N. Probing pocket depth (PPD) and clinical attachment loss (CAL) were measured at four sites. As a practically feasible procedure, probing pocket depth (PPD) and clinical attachment loss (CAL) were measured at four sites, not 6 sites, due to available time and resources. Our chosen definition of periodontitis was three or more PPDs of 5 mm or more on different teeth, third molars excluded [23,24]. The number of sites with PPD ≥4 mm and PPD >5 mm, and the number of teeth with CAL ≥4 mm was recorded.

Bleeding on probing (BOP) was measured and BOP% was calculated as the number of bleeding sites (n)/sites measured (n) × 100. Caries status was obtained in accordance with the DMFT system; decayed teeth (DT), missing teeth (MT), filled teeth (FT), and sound teeth (ST). FT is defined as the number of filled teeth that do not need treatment. These teeth possess a permanent filling or crown. For CAL and OPG measurements, a maximum of 32 teeth were included, but for the DMFT and PPD measurements counts from OPUS Dental, the maximum was 28 teeth because third molars were not counted. The results were recorded in the Opus Dental data program.

### 2.4. Blood Sampling

Blood samples were obtained by venipuncture between 8 a.m. and 9 a.m. after an overnight fast. The Vacuette serum, citrate, and EDTA tubes were from Greiner Bio-one GmbH (Frickenhausen, Germany). The EDTA tubes for plasma cytokine analysis were immediately placed on crushed ice and centrifuged at 1500× *g* for 15 min at +4 °C, and the plasma was stored at −80 °C until analysis.

### 2.5. Multiplex Immunoassay

Twenty-seven cytokines in EDTA plasma (P) were analyzed as described in a previous study using multiplex technology in a case-control study with 50 AIP cases; this previous study included the three edentulous cases that were excluded from the present study [11]. Furthermore, analysis of P-Insulin, P-C-peptide, P-Gastric inhibitory polypeptide, P-Visfatin, P-Resistin, P-Leptin, P-Ghrelin, and P-Plasminogen activator inhibitor-1, all given in pg/mL, were performed using the Bio-Plex 200 system (Bio-Rad) and a Bioplex Pro human diabetes immunoassay kit. Relevant quality control measurements were applied while running the assays.

### 2.6. Enzyme Immunoassay

Prothrombin fragment 1+2 (PTF1.2) (pmol/L) in EDTA plasma was analyzed by the Enzygnost F1+2 (monoclonal) kit (Dade Behring, Marburg GmbH, Marburg, Germany). The complement activation biomarkers P-C3bc and the soluble C5b-9 terminal complement complex were analyzed in EDTA plasma using ELISA as described previously and expressed as complement arbitrary units/mL (CAU/mL) [25]. To measure the optical density, we used a MRX microplate reader (Dynex Technologies, Denkendorf, Germany) [26].

### 2.7. Biochemistry Tests

Blood platelets, expressed as 10^9^/L, were analyzed on a Siemens ADVIA 2120 Hematology System (Siemens Healthcare Diagnostics Limited, Camberly, UK). The Tosoh G8 high-performance liquid chromatography (HPLC) was used to analyze B-HbA1c reported as mmol/mol. The ADVIA^®^ 1800 system (Siemens Medical Solutions Diagnostics, Japan) and reagents from Siemens Healthcare Diagnostics Limited was used to measure S-Bilirubin (µmol/L), S-carbamide (mmol/L), S-phosphate (mmol/L), S-gamma-glutamyl transferase GGT, U/L), S-calcium (mmol/L), S-Alkaline phosphatase (ALP, U/L), and other routine biochemistry tests earlier described [11]. U-epinephrine, U-norepinephrine, (both nmol/mmol creatinine), and U-vanillylmandelic acid (VMA, µmol/mmol creatinine) were analyzed in a morning spot urine by HPLC (Merck Hitachi) using kits from Chromsystems Instruments & Chemicals GmbH, (Munich, Germany). P-parathyroid hormone (PTH, pmol/L) was analyzed using ADVIA Centaur XP (Siemens Healthcare Diagnostics, Tarrytown, NY, USA). BN ProSpec^®^ nephelometer (Siemens Healthcare Diagnostics Ltd.) was used to measure the serum levels (g/L) of complement components C3 and C4, S-albumin, prealbumin, and alpha-1 antitrypsin. Urine porphobilinogen (U-PBG) levels and urine aminolevulinic acid (U-ALA) were analyzed using a kit from BioRad Laboratories (Munich, Germany) and expressed as µmol/mmol creatinine. Total porphyrins were analyzed as described previously and expressed as nmol/mmol creatinine [27].

### 2.8. Indexes, Scores, and Ratios

P-C3bc/C3 ratio (CAU/mL/g/L), S-Glucose/P-Insulin-ratio (mmol/L/pg/mL), and U-Albumin/creatinine (mg/mmol) ratio were calculated from other measured data. The liver fibrosis index 4 (FIB4) was calculated as follows: (age × AST)/(platelets × (sqr. (ALT)), with age in years, aspartate aminotransferase (AST) and alanine aminotransferase (ALT) as U/L, and platelet count as 10^9^/L. The estimated glomerular filtration rate (eGFR) was calculated using the Chronic Kidney Disease Epidemiology Collaboration (CKD-EPI) creatinine equation (mL/min/1.73 m^2^) [28].

An excel spreadsheet from the University of Oxford was used to estimate beta cell function, HOMA%B (%B), insulin sensitivity, HOMA%S (%S), and insulin resistance HOMA-IR (IR), based on serum glucose and plasma C-peptide.

### 2.9. Recording of Smoking, Diet, and Use of Statins

The AIP cases and controls were questioned regarding symptoms, diet, and smoking. Smoking was recorded in pack-years, which is defined as packs of 20 cigarettes smoked per day × number of years as a smoker. A 7-day diet logbook was used to obtain information on dietary intake of added sugar and carbohydrates (g per day). The diet logbook was scanned using the Teleform program version 6.0 (Datascan, Oslo, Norway). The daily intake was calculated using the food database and software system (KBS) from the Department of Nutrition, University of Oslo. Diet data were obtained from 43 cases and 43 matched controls.

### 2.10. Statistical Analysis

The Wilcoxon matched-pairs signed-rank test was used on the matched case-control data. While Spearman’s nonparametric correlation was used on case and control data, the Mann–Whitney U-test was used on non-matched data. Fischer’s exact test was performed for categorical variables. *p* < 0.05 was considered statistically significant. The statistical analysis was performed using Prism version 6.0 from GraphPad Software Inc. (San Diego, CA, USA).

## 3. Results

### 3.1. Baseline Characteristics

The baseline demographic characteristics of the AIP population and the controls were equal on most variables (Table 1). The number of DT, MT, and FT were not significantly different in the AIP cases versus the controls, although the median number of MT was three in the cases versus one in the controls (Table 1). Significantly more third molars were present in the cases (Table 1). The number of sites with elevated PPD, the BOP%, the number of sites with CAL, and the number of persons with periodontitis were not significantly different in the cases and the controls. Thirty percent of the AIP cases had periodontitis, and 21% of the controls (*p* = 0.48, Table 1). Among the symptomatic AIP cases, 9 of the 32 got comments on the OPG, and among the asymptomatic AIP cases, 2 of the 15 got comments on OPG (Table A1, Appendix A).

### 3.2. Sugar Intake, Glucose Metabolism, and Dental Health

The intake of added sugar in the AIP cases was not significantly higher than the controls (Table 1). However, there was a significantly higher intake of carbohydrates in the AIP cases with periodontitis versus the controls with periodontitis (Table 2). While several AIP cases reported that they sought out various forms of sugar intake as an everyday precaution against AIP attacks, an added sugar intake did not significantly correlate with the DT in either the AIP cases (r = 0.29, *p* = 0.06) or in the controls (r = −0.18, *p* = 0.24). PPD >5 mm was similarly not significantly correlated with the HbA1c in the cases (r = 0.19, *p* = 0.19) or the controls (r = 0.21, *p* = 0.16) or sugar intake in grams in either the AIP cases (r = −0.07, *p* = 0.66) or the controls (r = 0.02, *p* = 0.91). PPD >5 mm correlated positively with P-insulin (r = 0.32, *p* = 0.03) and P-C-peptide (r = 0.50, *p* < 0.001) in the AIP cases but not in the controls, PPD >5 mm vs. P-insulin (r = 0.28, *p* = 0.06) and PPD >5 mm vs. P-C-peptide (r = 0.23, *p* = 0.12).

P-Insulin and P-C-peptide were significantly higher in the AIP cases with periodontitis versus those without (Table 2). We found a lower %B in AIP cases (median 67%) vs. the controls (median 75%), (*p* = 0.005%), a higher %S (*p* = 0.02), and a lower IR (*p* = 0.04). The AIP cases with periodontitis (n = 14) had lower %S and higher IR than the AIP cases without periodontitis (n = 33) (*p* = 0.007). In the AIP cases, the %S correlated negatively with the number of PPD >5 mm (*p* < 0.001) and missing teeth (n) (*p* = 0.03). We found that %B in the AIP cases was significantly and negatively correlated with several cytokines in plasma, IL-5, IL-7, IL-10, IL-12, IL-15, CCL4, CCL11, GM-CSF, TNF (all *p* < 0.05), see Table A2, Appendix A.

### 3.3. Smoking and Dental Health

Smoking pack-years were slightly higher in the cases as compared to the controls, but the number of current smokers and former smokers were similar in the two groups (Table 1). Smoking pack-years correlated negatively with the number of teeth, ST, and positively with DMFT in the cases, but not in the controls (Table 3). Smoking pack-years correlated positively with MT both in the cases and the controls (Table 3). Simultaneously, PPD >5 mm correlated positively with smoking pack-years both in the controls and the cases (Table 4).

### 3.4. AIP Disease Activity Markers and Dental Health

The AIP disease markers U-PBG and U-ALA were significantly higher in the AIP cases compared to the controls (Table 1). U-PBG and U-ALA neither correlated with DMFT status (Table 3) nor with periodontitis markers (Table 4) in AIP cases. U-ALA correlated positively with apical periodontitis in the controls (Table 4).

### 3.5. Dental Health and Kidney Function

The number of ST and DMFT in both the cases and the controls correlated with the estimated glomerular filtration rate based on serum creatinine (eGFR), a marker of kidney function (Table 5). Similarly, MT and FT correlated negatively with eGFR in both the cases and the controls (Table 5). In the controls, CAL ≥4 mm correlated negatively with the eGFR and positively with serum carbamide and creatinine (Table 6). In these cases, both CAL ≥4 mm and PPD >5 mm correlated negatively with eGFR (Table 6). Apical periodontitis correlated positively with S-urate levels in the cases but not in the controls (Table 6).

Because secondary hyperparathyroidism from CKD could affect dental health, we also measured PTH levels. The number of teeth correlated negatively with parathyroid hormone (PTH) levels both in the cases and the controls (Table 5). MT correlated positively with PTH in the cases but not in the controls (Table 5). The AIP cases with eGFR <90 had significantly higher PTH levels, median 5.0 pmol/L (IQR 4.1–7.9), than AIP cases with normal kidney function, median 3.5 (IQR 2.6–5.5) (*p* = 0.04). S-ALP did not differ between the AIP cases with eGFR <90, ALP 73 U/L (62–85) vs. AIP cases with normal kidney function who had ALP 68 (62–92), *p* = 0.87, indicating no effects on the bone.

### 3.6. Dental Health and Liver Parameters

The number of teeth, ST, MT, FT, and DMFT correlated negatively with the liver fibrosis score FIB4 in both the cases and the controls (Table 5). CAL ≥4 mm correlated positively with FIB4 in the cases and in the controls (Table 6). S-bilirubin correlated negatively with ST and the number of teeth and positively with MT, FT, and DMFT in the cases, but not in the controls (Table 5). There was a significant difference in S-bilirubin levels between the cases, the median 10 (IQR 8–12), and the controls 12 (8–15), *p* = 0.04.

### 3.7. Cytokines and Dental Health

**Figure 1:**
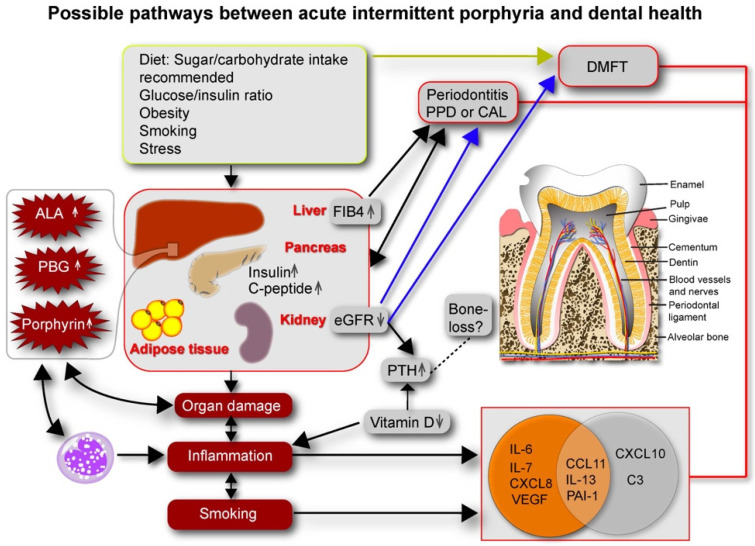
Possible pathways between acute intermittent porphyria (AIP) and dental health. Bård Ove Karlsen created Figure 1 in Adobe Illustrator version CC 2019 (23.0.0), Available online: https://www.adobe.com/products/illustrator.html (accessed on 10–27 June 2019).

AIP cases with periodontitis had similar cytokine levels compared to AIP cases without periodontitis (Table 7). Both AIP cases with and without periodontitis had higher cytokine levels compared to their corresponding controls (Table 7). CC motif chemokine ligand 11 (CCL11) and CXC motif chemokine 10 (CXCL10) correlated positively with MT, and CXCL10 correlated positively with DMFT in both the cases and the controls (Table 3). CXCL10 correlated negatively with ST both in the cases and the controls (Table 3). In the cases, but not the controls, interleukin (IL)-13 correlated positively with DT (Table 3 and Figure 1). In the controls but not the cases, interleukin-1 receptor antagonist (IL-1RA), IL-6, CXCL8, IL-13, and CCL11 correlated positively with DMFT (Table 3). In the cases, CAL ≥4 mm correlated positively with IL-13, and CCL11 and BOP% correlated negatively with plasma IL-1β, IL-1RA, and IL-7; correlations were not seen in the controls (Table 4).

ALA: delta-aminolevulinic acid; PBG: porphobilinogen; PPD: probing pocket depth; CAL: clinical attachment loss; DMFT: decayed missing filled teeth; PTH: parathyroid hormone; eGFR: estimated glomerular filtration rate; FIB4: liver fibrosis index 4; PAI-1: plasminogen activator inhibitor-1; IL-interleukins; CCL: chemokine ligands; CXCL: CXC-motif chemokine ligand; VEGF: vascular endothelial growth factor.

Sugar, which is used in the treatment of AIP-attacks, as well as the general recommendation of elevated carbohydrate intake in AIP, may affect dental health. We found that AIP cases with periodontitis had increased insulin and C-peptide, which is consistent with insulin resistance. The carbohydrate intake was higher in the AIP cases with periodontitis. The AIP-mediated end-organ damage of the kidneys and the liver and the associated chronic inflammation, possibly due to the porphyrin precursors delta aminolevulinic acid (ALA) and porphobilinogen (PBG) and in some cases due to smoking, might all worsen dental health. We found that a reduced estimated glomerular filtration rate (eGFR) due to kidney damage and increased liver fibrosis score 4 (FIB4) due to liver disease were associated with poor dental health. Organ damage might activate inflammation and stimulate cytokine release. In our study, the levels of C-C motif chemokine ligand (CCL)11, interleukin- 6, -7, and (IL)-13, VEGF (vascular endothelial growth factor), plasminogen activator inhibitor-1 (PAI-1), and C-X-C motif chemokine ligand (CXCL)8 were correlated with smoking (orange circle), and smoking pack-years was correlated with periodontitis markers. In the lighter right part of the orange circle that is merged with the grey circle, the three markers, which all correlate with smoking pack-years, also correlate with dental markers: CCL11 vs. MT and CAL ≥4, IL-13 vs. DT and CAL ≥4, and PAI-1 vs. PPD >5. CXCL10 and complement C3 (grey circle) were correlated with DMFT status and PPD, respectively. AIP-induced end-organ damage, particularly in the kidneys, and inflammation due to poor dental health, may possibly increase AIP disease activity.

### 3.8. Complement Markers and Dental Health

PPD >5 mm correlated positively with complement C3 levels in both the cases and the controls (Table 4). The terminal complement complex (TCC) correlated positively with DMFT in the controls but not in the cases (Table 3). While PPD ≥4 mm correlated negatively with C3bc/C3 in the cases; the correlation was positive in the controls (Table 4). In the controls, BOP% correlated positively with C3bc/C3 (Table 4).

### 3.9. Prothrombin F1+2, Plasminogen Activator Inhibitor-1, and Dental Health

The coagulation marker PTF1.2 correlated negatively with the number of teeth in the controls, but not in the cases (Table 3). PTF1.2 correlated positively with the number of filled teeth in the AIP cases, but not in the controls (Table 3). In the controls but not in the cases, PAI-1 correlated negatively with ST and positively with DT, FT, and DMFT (Table 3). In the AIP cases, PAI-1 correlated positively with PPD >5 mm (Table 4).

## 4. Discussion

This study demonstrates that AIP patients with the AIP-related complication kidney failure or liver fibrosis had worsened dental health as determined by DMFT and CAL ≥4. The frequency of periodontitis and DMFT status was not different in the AIP cases versus the controls. Insulin and C-peptide were higher in AIP cases with periodontitis versus those without. While the biochemical disease activity biomarkers U-PBG and ALA did not correlate with dental health in AIP cases, these markers only describe AIP disease activity at one moment in time and not over months or years. Similar to the previous findings, the levels of several cytokines were higher in the AIP cases compared to the controls [8].

Surprisingly, we did not find more DT in the AIP cases versus the controls despite a slightly higher sugar intake in the cases [29]. Because AIP cases receive reimbursement for dental treatment in Norway, it might have muted the observed effect of AIP on dental health. However, in AIP cases with periodontitis, the carbohydrate intake was significantly higher than those without periodontitis, suggesting that diet plays a role in the development of periodontitis in AIP. Because the mean age of AIP patients with periodontitis was ten years higher than those without periodontitis in our study, a confounding effect of aging on this association cannot be excluded.

There are mechanistic links between periodontitis and other metabolic diseases, namely diabetes, which results in the production of inflammatory cytokines such as IL-1β, TNF, IL-6, as well as oxidative stress [16]. The cytokine elevation seen in AIP cases is possibly a result of the neurotoxic porphyrin precursors ALA and PBG inducing organ damage in the liver and kidney [11,30,31]. In our previous study, we observed disturbed insulin signaling in the symptomatic AIP cases [29]. As hyperglycemia and obesity are linked to metabolic inflammation and periodontitis, we speculate that AIP might be one of the many susceptibility factors for periodontitis, a susceptibility factor that is possibly mediated by chronic inflammation, kidney failure, and a high sugar intake [32].

In this study, we found significant correlations between smoking pack-years, periodontitis, and inflammation. Because we observed stronger correlations between smoking and inflammation in the AIP cases versus the controls, this might suggest that smoking has a greater effect on dental health in the AIP cases compared to the controls. We speculate that the combination of smoking and AIP might lead to more inflammation and organ damage than smoking alone. Nonetheless, in both the cases and the controls, PPD >5 mm correlated highly with smoking pack-years. It is well-established that cardiovascular risks are associated with the incidence and prevalence of periodontal disease and with changes in smoking rate [33,34]. Another study identified CCL2, CCL11, years of smoking, and age as periodontitis associated factors [35]. While smoking avoidance is important for periodontal health, smoking avoidance might be especially essential in AIP.

The AIP biochemical disease markers ALA and PBG did not correlate with dental health in the AIP cases, which might suggest that the biochemical disease activity does not directly affect dental health. Although ALA and PBG were analyzed only at one time point, a previous study investigating the biological variation of U-ALA and U-PBG in AIP suggests that AIP patients vary around their own typical porphyrin precursor level [36]. Nonetheless, end-organ effects of AIP such as kidney and liver damage might be better indicators of AIP disease activity over time compared to single measurements of ALA and PBG. Both poor dental health and reduced kidney function might possibly worsen AIP disease activity as chronic inflammation and reduced kidney function might increase AIP disease activity (Figure 1) [37].

We found significant correlations between MT, FT, CAL ≥4 mm, and biomarkers of kidney function in both the cases and the controls. However, only in AIP cases was there any significant correlation between PPD >5 mm and kidney function. Furthermore, AIP cases with eGFR <90 had significantly higher PTH levels than the AIP cases with normal kidney function. Increased PTH levels also correlated with MT. These associations suggest a possible mechanism of chronic kidney disease-mediated dental health impairment in AIP because decreased renal function might affect tooth loss through secondary hyperparathyroidism, which increases calcium loss from the bone. Our observation that increased PTH levels correlates with reduced kidney function in AIP fits with a recent review describing a non-direct link between chronic kidney diseases and periodontitis [38]. While increased PTH might be due to reduced kidney function or insufficient 1,25-dihydroxy vitamin D levels, the PTH elevation observed in AIP patients with CKD in this study was likely mediated by kidney disease itself because our previous study demonstrated that vitamin D deficiency is not different between the AIP cases and the controls [29]. However, vitamin D levels are important for oral health, and low vitamin D levels might also be associated with enhanced inflammation [39,40,41].

MT, ST, and FT correlated with the liver fibrosis marker FIB4 both in the cases and the controls. We found significant correlations between liver fibrosis markers and periodontitis markers, especially in the AIP cases. Such associations have been found in patients with HCV or HBV infection in a study that stressed the importance of controlling oral disease for the prevention and management of liver fibrosis [42]. Although AIP itself is not associated with liver fibrosis, alcohol intake and other diseases such as fatty liver might contribute to liver fibrosis.

Surprisingly, AIP cases with periodontitis did not have significantly higher cytokine levels compared to AIP cases without periodontitis. One possible explanation might be that the additional inflammation of periodontitis did not contribute significantly to the already present low-grade inflammation in AIP. In line with our previous study, we found that AIP cases had higher cytokine levels compared to their controls, probably not due to an increased incidence of other inflammatory diseases in AIP [11]. Inflammatory conditions such as rheumatoid arthritis are known to be associated with periodontal conditions [43]. We previously reported elevated cytokines including IL-17 in AIP with a cluster analysis, suggesting a T-helper type 17 inflammatory response similar to the findings in periodontal lesions [11,44]. We found that DMFT data correlated significantly with the chemokine CXCL10 in both the cases and the controls, indicating that more missing teeth and diseased teeth are associated with inflammation.

Local complement dysregulation is present in periodontitis [14,15]. In our previous study, both low-degree complement activation and correlations between U-PBG and the terminal complement complex (TCC) were found in AIP [11]. In the present study, we found a positive correlation between PPD >5 mm and complement C3 in both AIP cases and the controls, which is likely related to inflammation. PPD >5 mm did not correlate with complement activation markers C3bc/C3 or TCC in either the AIP cases or the controls possibly because local complement activation in periodontitis might not always be measurable in plasma.

In our study, PAI-1 was significantly correlated with DT, FT, ST, DMFT, and apical periodontitis in the controls and to PPD >5 mm and smoking pack-years in the AIP cases. PAI-1, which is an inhibitor of the endogenous fibrinolytic system, is also known as an adipocytokine. It is related to a high BMI and other functions associated with insulin resistance and type 2 diabetes [45,46]. While the significance of the differential effect of PAI-1 on dental health in the cases and the controls is unclear, our data suggest that dental health might affect the fibrinolytic system.

We found, using HOMA scores, that the beta cell function and insulin resistance was lower in the cases compared to the matched controls. In the AIP cases, a lower beta cell function was associated with inflammation. Periodontitis in AIP was associated with a higher insulin resistance.

A strength of this study is that it provides a novel overview of dental health in AIP, including the association of dental health with various biochemical markers. The association of dental health has not previously been systematically examined in AIP. Another strength is the relatively high number of participants for a very rare disease. A limitation is that dental hygiene habits, including the use of fluoride and snuff, was not recorded and previous dental records were not available. There is also a relatively small number of participants in this study, and there is a relatively large number of statistical tests performed. This could have led to false-positive results. Our lack of power calculations regarding dental health in AIP, since there were no data on expected dental health in AIP in the literature, in combination with the rarity of the disease, may have led to low power that may have reduced the chance of detecting a true effect. It might also have reduced the likelihood that our statistically significant results were actually reflecting a true effect. Most participants had the same AIP mutation and were from Norway. This may affect the external validity, but on the other hand, the same mutation is also prevalent in Sweden and all the AIP mutations lead to a reduced function of the same enzyme.

## 5. Conclusions

The carbohydrate intake was significantly higher in AIP cases with periodontitis compared to the controls with periodontitis. Simultaneously, insulin and C-peptide levels were significantly higher in the AIP cases with periodontitis versus the AIP cases without. Periodontitis in AIP was also associated with a higher insulin resistance. In cases with AIP, a lower beta cell function was associated with inflammation. Decreased renal function in AIP was associated with an increased number of MT and increased PTH. In the AIP cases, liver and kidney markers, smoking status, and inflammatory markers were correlated with reduced dental health. These results suggest that AIP-associated organ complications and chronic low-grade inflammation may worsen dental health, although due to the small sample size, the possibility of false positive findings cannot be excluded. Bearing in mind that the results from small subgroups could be less reliable, we anyhow cautiously suggest a hypothesis that inflammation induced by poor dental health in some cases might in turn worsen AIP.

## Figures and Tables

**Table 1:** Baseline demographic characteristics of the study population.

	**Controls**	**AIP ^a^ Cases**	**OR (CI)**	***p***
Age, years (SD)	48.9 (18.1)	49.0 (17.8)		0.51
Height, cm (SD)	172.0 (9.6)	172.0 (9.8)		0.90
Weight, kg (SD)	80.5 (14.4)	81.3 (15.7)		0.65
Body mass index, kg/m^2^ (SD)	27.2 (4.0)	27.3 (3.9)		0.89
Waist/hip ratio	0.97 (0.90–1.01)	0.97 (0.88–1.02)		0.70
Women, n (%)	20 (42.6%)	20 (42.6%)		
Men, n (%)	27 (57.4%)	27 (57.4%)		
Symptomatic AIP ^a^, n (%) Asymptomatic AIP ^a^, n (%)		32 (68.1%), 15 (31.9%)		
*Disease activity and dental health:*				
U-PBG ^a^, μmol/mmol creatinine	0.4 (0.3–0.5)	2.5 (0.9–7.3)		**<0.001**
U-ALA ^a^, μmol/mmol creatinine	1.9 (1.6–2.3)	3.8 (2.4–7.7)		**<0.001**
U-Total porphyrins, nmol/mmol creatinine	82 (34–123)	202 (96–659)		**<0.001**
Sound, healthy teeth, n	9.0 (5–18)	10.0 (5–17)		0.91
Decayed teeth, n	1.0 (0–3)	2.0 (0–4)		0.34
Missing teeth, n	1.0 (0–5)	3.0 (0–5)		0.74
Filled teeth, n	12.0 (6–16)	10.0 (7–16)		0.67
DMFT ^b^, n	19.0 (9–22)	18.0 (11–23)		0.77
Sites (n) with PPD ^c^ > 5 mm	0.0 (0.0–0.0)	0.0 (0.0–1.0)		0.22
Bleeding on probing ^c^, %	8.0 (2.0–12.0)	7.0 (4.0–12.0)		0.96
Sites (n) with CAL ^c^ ≥ 4 mm	9.0 (3.0–18.5)	10.0 (2.0–22.0)		0.25
Periodontitis ^d^	10 (21%)	14 (30%)	1.6 (0.6–4.0)	0.48
Teeth, n	27 (24–28)	27 (23–30)		0.40
Missing teeth from OPG ^e^, n	5.0 (3.0–8.0)	4.0 (1.0–9.0)		0.32
Apical periodontitis from OPG ^e^, n	0.0 (0.0–1.0)	0.0 (0.0–1.0)		0.79
Wisdom tooth present from OPG ^e^, n	1.0 (0.0–3.0)	2.0 (0.0–3.0)		**0.03**
Difficulties chewing, current, n (%)	2 (4.3%)	2 (4.3%)	1.00	1.00
Dryness of mouth, ever, n (%) persons	2 (4.3%)	8 (17.0%)		0.09
Smoking pack-years ^b^	0.4 (0.0–5.8)	1.8 (0.0–12.3)		**0.045**
Never smokers, n (%)	17 (36%)	18 (38%)		
Former smokers, n (%)	24 (51%)	19 (40%)	0.75 (0.31–1.83)	0.65
Current smokers, n (%)	6 (12%)	10 (20%)	1.57 (0.47–5.28)	0.55
Ethanol intake, g per day	6.5 (0–15.7)	4.2 (0–13.5)		0.34
Carbohydrate intake, g/day	191 (157–210)	202 (166–264)		0.11
Added sugar intake, g/day	36 (27–50)	43 (33–58)		0.18
Diabetes mellitus, n persons (%)	3 (6.4%)	4 (8.5%)	1.36	1.00
B-HbA1c, mmol/mol	34 (31–38)	36 (34–39)		**0.02**
P-Insulin, pg/mL	35 (17–99)	23 (3.4–46)		**0.04**
P-C-peptide, pg/mL	1177 (944–1624)	981 (725–1322)		**0.03**
HOMA % B ^f^	75 (66–89)	67 (49–80)		**0.005**
HOMA % S ^f^	117 (85–142)	135 (103–190)		**0.02**
HOMA IR ^f^	0.86 (0.71–1.2)	0.74 (0.53–0.97)		**0.04**
P-Prothrombin F1+2 ^b^, pmol/L	153 (128–211)	186 (140–288)		**0.002**

The data represent the mean values (SDs), n (%), or median values and IQR from 47 acute intermittent porphyria (AIP) cases and 47 matched controls; however, the diet data are from 43 matched pairs. The Wilcoxon matched pairs signed rank test was used for most of the data. Statistically significant differences are indicated in bold. Fisher’s exact test and OR, odds ratio with CI, 95% confidence interval, was used for smokers and those with difficulty chewing. CI was not calculated when n < 6. OR for smoking was calculated by comparing former smokers against never-smokers in cases versus controls and current smokers against never-smokers in cases versus controls. The *p* values are exact two-tailed p. ^a^ Acute intermittent porphyria (AIP) disease activity markers; U-PBG: urine porphobilinogen, ALA, urine delta aminolevulinic acid. ^b^ Markers of dental health: Sum of decayed teeth; missing teeth; and filled teeth. ^c^ Markers of marginal periodontitis probing pocket depth, (PPD), bleeding on probing (BOP%), and clinical attachment loss (CAL). ^d^ Periodontitis defined as three or more PPDs of 5 mm or more on different teeth, third molars excluded. ^e^ Orthopantogram. ^f^ Homeostasis model assessment (HOMA) scores for beta cell function (HOMA %B), insulin sensitivity, (HOMA %S) and insulin resistance (HOMA-IR) based on serum glucose and plasma (P) C-peptide.

**Table 2:** Diet and glucose metabolism markers in AIP ^a^ cases with and without periodontitis compared to controls.

	**Ctrl** **(n = 37)**	**Case** **(n = 33)**	**Ctrl − PD ^b^** vs. **Case − PD ^b^**	**Ctrl** **(n = 10)**	**Case** **(n = 14)**	**Ctrl + PD vs.** **Case + PD**	**Case − PD vs.** **Case + PD**	**Ctrl − PD vs.** **Ctrl + PD**
Periodontitis	−	−	*p*	+	+	*p*	*p*	*p*
S-Vitamin D ^c^	56 (39–73)	58 (42–71)	0.76	58 (33–72)	53 (41–76)	0.92	0.93	0.92
S-Cobalamine ^d^	317 (237–372)	384 (305–439)	**0.01**	308 (266–332)	277 (237–342)	0.48	**0.002**	0.85
S-Ferritin, μg/L	142 (45–196)	102 (43–191)	0.47	109 (49–147)	176 (98–338)	**0.004**	0.10	0.53
S-Triglyceride ^e^	1.0 (0.8–1.4)	1.2 (1.0–1.6)	0.06	1.1 (0.7–1.9)	1.8 (1.0–3.0)	0.07	0.11	0.73
S-Total cholesterol ^e^	5.1 (4.4–5.7)	5.7 (4.9–6.3)	**0.04**	5.4 (4.6–5.7)	5.5 (5.1–6.8)	0.17	0.62	0.53
P-GIP ^f^, pg/mL	72 (32–144)	12 (6.8–38)	**<0.001**	38 (25–62)	78 (20–112)	0.28	**0.007**	0.11
P-Visfatin, pg/mL	662 (440–887)	813 (569–1648)	0.26	598 (458–878)	1299 (656–2290)	**0.02**	0.13	0.77
P-Resistin, pg/mL	3307 (2649–4154)	2598 (2254–4103)	**0.04**	3177 (2388–4243)	3163 (2259–3623)	0.89	0.40	0.75
P-Leptin pg/mL	2686 (798–8571)	2802 (1183–9319)	0.64	8335 (2927–14,882)	4505 (2049–9036)	0.40	0.22	**0.03**
P-Ghrelin, pg/mL	1266 (866–1548)	1259 (989–1632)	0.98	913 (705–1538)	946 (706–1254)	0.89	0.052	0.12
P-Insulin, pg/mL	35 (16–91)	12 (2.8–33)	**0.002**	45 (25–160)	42 (21–81)	0.75	**0.007**	0.46
S-Glucose/P-Insulin ^g^	0.2 (0.1–0.3)	0.5 (0.2–1.9)	**0.002**	0.1 (0.04–0.2)	0.1 (0.1–0.2)	0.67	**0.007**	0.49
P-C-peptide, pg/mL	1116 (838–1554)	879 (695–1126)	**0.02**	1388 (1160–1745)	1353 (1080–1692)	0.98	**0.006**	0.18
HOMA % B ^h^	75 (63–90)	64 (49–77)	**0.008**	75 (71–88)	75 (49–92)	0.63	0.22	0.62
HOMA % S ^h^	119 (87–168)	149 (120–202)	**0.03**	97 (73–117)	100 (71–126)	0.97	**0.007**	0.15
HOMA IR ^h^	0.84 (0.60–1.1)	0.67 (0.50–0.83)	**0.03**	1.0 (0.86–1.4)	1.0 (0.8–1.4)	0.98	**0.007**	0.15
B-HbA1c ^i^	34 (30–37)	37 (33–39)	**0.02**	37 (36–39)	34 (34–42)	0.49	0.74	**0.03**
Added sugar ^j^	37 (28–50)	42 (26–66)	0.60	31 (19–46)	48 (35–53)	0.09	0.70	0.22
Carbohydrates ^j^	195 (175–235)	203 (164–268)	0.59	155 (109–190)	201 (179–210)	**0.02**	0.82	**0.009**
S-TSH, IU/L	1.7 (1.2–2.2)	1.6 (1.2–2.3)	0.71	2.1 (1.4–2.8)	2.4 (1.9–3.1)	0.31	**0.002**	0.30
U-VMA ^k^	1.6 (1.1–2.0)	1.0 (0.7–1.7)	**0.004**	1.8 (1.2–2.2)	2.0 (1.1–2.5)	0.63	**0.02**	0.70

The data represent the median values and IQR from 47 AIP cases and 47 matched controls, but for diet logbook data n = 43, of which Ctrl − PD n = 33, Case − PD n = 30, Ctrl + PD n = 10, and Case + PD n = 13. Mann–Whitney U-test was used. *p* values are exact, two-tailed p. Statistically significant differences are indicated in bold. ^a^ Acute intermittent porphyria. ^b^ Periodontitis. ^c^ S-25-OH Vitamin D, nmol/L. ^d^ pmol/L. ^e^ mmol/L. ^f^ Gastric inhibitory polypeptide. ^g^ S-glucose/P-insulin-ratio, mmol/L/pg/mL. ^h^ Homeostasis model assessment (HOMA) score. ^i^ mmol/mol. ^j^ grams. ^k^ Vanillylmandelic acid, μmol/mmol.

**Table 3:** Correlations between DMFT data and AIP disease activity markers, complement activation markers, coagulation markers, inflammatory markers, and smoking status.

	Teeth ^f^		ST ^f^		DT ^f^		MT ^f^		FT ^f^		DMFT ^f^		Pack-Years	
	Ctrl.	Case	Ctrl.	Case	Ctrl.	Case	Ctrl.	Case	Ctrl.	Case	Ctrl.	Case	Ctrl.	Case
U-PBG ^a^	−0.42 **	−0.13	−0.46 **	−0.17	0.18	−0.01	0.37 **	0.08	0.28	0.21	0.46 **	0.19	0.29	0.19
U-ALA ^a^	−0.06	0.03	−0.24	0.04	0.10	−0.01	0.02	−0.07	0.29	0.04	0.25	−0.02	0.01	0.18
U-Porph ^a^	−0.07	−0.07	−0.08	0.05	−0.23	0.04	0.11	−0.04	0.01	−0.04	0.04	−0.05	0.10	0.17
C3bc/C3 ^b^	0.02	0.01	−0.07	0.11	0.04	−0.08	0.04	−0.06	0.13	−0.13	0.07	−0.10	−0.14	−0.08
C3 ^b^	−0.13	0.16	−0.13	0.12	0.16	0.16	0.10	−0.11	0.06	−0.21	0.15	−0.14	0.26	−0.06
TCC ^b^	−0.19	−0.18	−0.26	−0.24	0.08	−0.14	0.27	0.18	0.22	0.16	0.29 *	0.25	0.13	0.14
PTF1.2 ^c^	−0.29 *	−0.21	−0.25	−0.26	−0.02	−0.08	0.16	0.24	0.20	0.37 *	0.22	0.28	0.39 **	0.22
PAI-1 ^c^	−0.17	−0.21	−0.50 ***	−0.21	0.35 *	0.27	0.24	0.25	0.35 *	0.10	0.51 ***	0.22	0.07	0.41 **
IL-1β ^d^	−0.14	−0.08	−0.12	−0.08	−0.13	0.16	0.21	0.22	−0.06	−0.03	0.11	0.11	0.06	0.28
IL-1RA ^d^	−0.25	−0.02	−0.29 *	−0.04	−0.06	0.14	0.21	0.17	0.19	−0.04	0.31 *	0.06	0.21	0.29
IL-6 ^d^	−0.26	−0.06	−0.31 *	−0.06	0.04	0.16	0.31 *	0.21	0.05	−0.04	0.32 *	0.08	0.23	0.40 **
IL-7 ^d^	−0.25	−0.13	−0.12	−0.06	−0.11	0.04	0.23	0.25	−0.15	0.00	0.09	0.08	−0.07	0.44 **
CXCL8 ^d^	−0.41 **	−0.13	−0.32 *	−0.20	−0.06	0.06	0.43 **	0.25	0.03	0.08	0.31 *	0.23	0.11	0.31 *
IL-13 ^d^	−0.26	−0.05	−0.38 *	−0.09	0.19	0.43 **	0.34 *	0.23	0.21	−0.08	0.38 **	0.10	0.36 **	0.30 *
CCL11 ^d^	−0.35 *	−0.15	−0.47 ***	−0.22	0.07	0.10	0.39 **	0.29 *	0.22	0.14	0.47 ***	0.24	0.44 **	0.44 **
CXCL10 ^d^	−0.28	−0.20	−0.30 *	−0.32 *	0.29 *	0.14	0.39 **	0.33 *	−0.03	0.25	0.32 *	0.33 *	0.16	0.18
VEGF ^d^	−0.13	−0.07	−0.23	−0.07	0.12	0.21	0.27	0.23	0.03	−0.04	0.24	0.10	0.15	0.31 *
Packyears ^e^	−0.23	−0.34 *	−0.25	−0.33 *	0.09	0.16	0.32 *	0.37 *	0.09	0.23	0.26	0.33 *		

The data represent the Spearman correlation coefficient r analyzed using the non-parametric Spearman correlation. Significant correlations are indicated in bold. The dental variables arise from 47 acute intermittent porphyria (AIP) cases and 47 controls. The *p* values are exact, two-tailed. * *p* < 0.05, ** *p* < 0.01, *** *p* < 0.001. All markers in the table are further described in Materials and Methods. ^a^ Acute intermittent porphyria (AIP) disease activity markers; U-PBG: urine porphobilinogen, ALA, urine delta aminolevulinic acid; U-Porph: urine total porphyrins. ^b^ Complement factor C3 and complement activation markers: C3bc/C3 and terminal complement complex (TCC). ^c^ Coagulation markers prothrombin F1+2 (PTF1.2) and plasminogen activator inhibitor-1 (PAI-1). ^d^ IL-interleukins, CCL—chemokine ligands, CXCL-CXC-motif chemokine ligand, VEGF-vascular endothelial growth factor. ^e^ Smoking pack-years. ^f^ Markers of dental health: Teeth, number of teeth; ST, sound teeth; DT, decayed teeth; MT, missing teeth; DMFT, sum of DT, MT, and FT.

**Table 4:** Correlations between periodontal health parameters versus AIP disease activity markers, complement activation markers, coagulation markers, inflammatory markers, and smoking status.

	CAL ^f^ ≥4		PPD ^f^ ≥4		PPD ^f^ >5		BOP ^f^ %		Apical Perio ^f^ OPG	
	Ctrl.	Case	Ctrl.	Case	Ctrl.	Case	Ctrl.	Case	Ctrl.	Case
U-PBG ^a^, µmol/mmol creat	0.21	−0.05	0.18	−0.19	0.06	−0.09	−0.27	0.08	0.19	−0.23
U-ALA ^a^, µmol/mmol creat	0.12	0.00	0.12	−0.16	0.04	−0.15	−0.21	−0.01	0.29 *	−0.20
U- Porph ^a^, nmol/mmol cr.	−0.27	−0.11	−0.14	−0.22	−0.04	−0.11	−0.25	0.01	−0.13	−0.24
C3bc/C3 ^b^, CAU/mL/g/L	0.17	−0.23	0.33 *	−0.31 *	−0.05	−0.20	0.45 **	0.02	0.08	−0.19
C3 ^b^, g/L	0.07	−0.01	0.30 *	0.26	0.31 *	0.34 *	0.05	0.11	0.05	0.19
TCC ^b^, CAU/mL	0.12	−0.02	0.21	−0.03	0.18	0.10	−0.20	0.15	−0.05	0.04
PTF1.2 ^c^, pmol/L	0.15	0.17	0.21	−0.02	0.31 *	−0.04	0.11	0.01	0.18	−0.04
PAI-1 ^c^, pg/mL	0.11	0.06	0.08	0.19	0.20	0.42 **	−0.04	−0.05	0.40 **	0.16
IL-1β ^d^, pg/mL	−0.02	0.17	0.02	0.02	−0.09	−0.05	0.22	−0.31 *	0.04	0.11
P-IL-1RA ^d^, pg/mL	0.05	0.25	0.11	0.02	0.02	−0.05	0.06	−0.30 *	0.10	0.06
IL-6 ^d^, pg/mL	0.16	0.29	0.26	0.04	0.09	0.02	0.14	−0.26	0.14	0.07
IL-7 ^d^, pg/mL	−0.09	0.24	−0.11	−0.18	−0.21	−0.14	0.00	−0.31 *	−0.01	−0.04
CXCL8 ^d^, pg/mL	0.06	0.29	0.12	0.04	0.04	0.04	0.03	−0.22	0.04	0.08
IL-13 ^d^, pg/mL	0.00	0.31 *	0.07	0.22	0.06	0.25	0.11	−0.15	−0.07	0.33 *
CCL11 ^d^, pg/mL	0.25	0.31 *	0.33 *	0.03	0.09	−0.01	0.17	−0.17	0.16	0.01
CXCL10 ^d^, pg/mL	0.15	0.07	0.35 *	−0.06	0.15	−0.14	−0.09	−0.15	−0.02	0.05
VEGF ^d^, pg/mL	−0.05	0.24	−0.01	0.04	0.06	−0.03	0.23	−0.23	0.05	0.07
Pack-years ^e^	0.19	0.33 *	0.41 **	0.17	0.57 ***	0.36 *	0.02	−0.12	−0.04	0.17

The data represent the Spearman correlation coefficient r analyzed using the non-parametric Spearman correlation. Significant correlations are indicated in bold. The dental variables arise from 47 acute intermittent porphyria (AIP) cases and 47 controls. For CAL measurements n = 45 because two AIP cases had crowns covering their natural teeth, which made it impossible to measure CAL. The *p* values are exact, two-tailed. * *p* < 0.05, ** *p* < 0.01, *** *p* < 0.001. All markers in the table are further described in Materials and Methods. ^a^ Acute intermittent porphyria (AIP) disease activity markers; U-PBG: urine porphobilinogen, ALA, urine delta-aminolevulinic acid; U-Porph: urine total porphyrins. ^b^ Complement factor C3 and complement activation markers: C3bc/C3 (complement arbitrary units (CAU), CAU/mL/g/L) and terminal complement complex (TCC, CAU/mL). ^c^ Coagulation markers prothrombin F1+2 (PTF1.2) and plasminogen activator inhibitor-1 (PAI-1). ^d^ IL-interleukins, CCL-chemokine ligands, CXCL-CXC-motif chemokine ligand, VEGF-vascular endothelial growth factor. ^e^ Smoking pack-years. ^f^ Markers of marginal periodontitis clinical attachment loss (CAL) and probing pocket depth, (PPD), bleeding on probing (BOP%), and from OPG apical periodontitis.

**Table 5:** Correlations between DMFT data and kidney function, liver function, and catecholamine parameters.

	Teeth ^l^		ST ^l^		DT ^l^		MT ^l^		FT ^l^		DMFT ^l^	
	Ctrl.	Case	Ctrl.	Case	Ctrl.	Case	Ctrl.	Case	Ctrl.	Case	Ctrl.	Case
Sodium, mmol/L	0.04	0.16	−0.31 *	0.20	0.06	−0.13	0.05	−0.25	0.37 **	−0.01	0.32 *	−0.21
Potassium, mmol/L	0.05	0.16	−0.11	0.11	−0.05	−0.19	0.07	−0.17	0.09	0.00	0.11	−0.11
Calcium, mmol/L	−0.02	0.06	−0.04	−0.10	−0.08	−0.01	0.12	−0.12	−0.04	0.14	0.05	0.09
PTH ^a^, pmol/L	−0.32 *	−0.48 ***	−0.30 *	−0.27	−0.04	−0.02	0.20	0.53 ***	0.25	0.14	0.29	0.27
eGFR Creatinine ^b^	0.58 **	0.51 ***	0.68 ***	0.49 ***	−0.19	0.09	−0.56 ***	−0.50 ***	−0.39 **	−0.49 ***	−0.67 ***	−0.51 ***
Urate, μmol/L	0.01	−0.28	0.00	−0.40 **	−0.12	0.20	0.12	0.31 *	0.03	0.36 *	0.02	0.39 **
Carbamide, mmol/L	−0.21	−0.32 *	−0.24	−0.29	0.27	−0.15	0.34 *	0.26	−0.03	0.32 *	0.25	0.30 *
Phosphate, mmol/L	−0.01	0.01	0.10	0.16	0.01	−0.27	−0.17	−0.14	−0.08	0.01	−0.13	−0.14
Creatinine, µmol/L	−0.04	−0.20	−0.11	−0.27	−0.02	0.13	0.15	0.24	0.06	0.28	0.09	0.28
Albumin, g/L	0.27	0.25	0.35 *	0.19	0.04	0.00	−0.26	−0.21	−0.19	−0.23	−0.35 *	−0.22
U-Albumin/creat. ^c^	0.14	−0.17	0.04	−0.10	0.11	0.17	−0.14	0.22	0.08	−0.07	−0.03	0.10
AAT ^d^, g/L	−0.16	−0.35 *	−0.01	−0.25	0.03	0.05	0.05	0.32 *	−0.20	0.02	0.02	0.27
Bilirubin, µmol/L	0.20	−0.37 *	0.07	−0.42 **	−0.13	−0.06	−0.06	0.33 *	−0.07	0.42 **	−0.07	0.43 **
GGT ^e^, U/L	−0.10	−0.03	−0.37 *	−0.05	0.16	0.22	0.34 *	0.07	0.13	−0.01	0.37 *	0.04
ALP ^f^, U/L	−0.09	−0.03	−0.12	−0.05	0.13	0.44 **	−0.01	0.04	−0.1	−0.19	0.13	0.05
Prealbumin, g/L	−0.08	0.13	−0.09	0.08	−0.09	0.12	0.22	−0.05	0.15	−0.21	0.08	−0.09
AST ^g^, IU/L	−0.02	−0.10	−0.24	−0.29 *	0.17	0.02	0.09	0.10	0.25	0.27	0.26	0.30 *
ALT ^h^, IU/L	0.14	0.08	−0.07	−0.05	0.17	0.30 *	0.03	0.01	0.08	−0.04	0.09	0.03
FIB4 ^i^	−0.53 ***	−0.55 ***	−0.69 ***	−0.59 ***	0.17	−0.10	0.58 ***	0.53 ***	0.53 ***	0.54 ***	0.69 ***	0.61 ***
U-Epinephrine ^j^	0.14	0.20	0.03	0.02	0.04	0.21	−0.08	−0.19	0.15	−0.19	−0.02	−0.03
U-Norepinephrine ^j^	−0.48 ***	−0.32 *	−0.44 **	−0.35 *	0.32 *	−0.04	0.43 **	0.26	0.16	0.25	0.44 **	0.37 *
U-VMA ^k^	−0.26	−0.17	−0.22	0.03	0.17	−0.10	0.19	0.07	0.08	−0.04	0.20	−0.01
IgG, g/L	−0.14	0.27	−0.06	0.21	0.06	−0.08	0.21	−0.25	−0.03	0.02	0.09	−0.20
IgA, g/L	−0.17	−0.04	−0.25	−0.29 *	0.21	0.40 **	0.28	0.11	0.09	0.17	0.28	0.29
IgM, g/L	−0.03	−0.11	0.16	−0.03	0.02	−0.06	−0.02	0.10	−0.25	−0.16	−0.17	0.02

The data represent the Spearman correlation coefficient analyzed using the non-parametric Spearman correlation. Significant correlations are indicated in bold. The dental variables arise from 47 acute intermittent porphyria (AIP) cases and 47 matched controls. All the biomarkers in the first column are serum or plasma except those marked with U for urine. The *p* values are exact, two-tailed. * *p* < 0.05, ** *p* < 0.01, *** *p*< 0.001. ^a^ Parathyroid hormone. ^b^ eGFR creatinine = relative estimated glomerular filtration rate (eGFR) calculated using the Chronic Kidney Disease Epidemiology Collaboration (CKD-EPI) with creatinine equation (mL/min/1.73 m^2^). ^c^ U-albumin/creatinine (mg/mmol). ^d^ S-alpha-1-antitrypsin. ^e^ Gamma-glutamyl transferase. ^f^ S-alkaline phosphatase. ^g^ S-AST-aspartate aminotransferase. ^h^ S-alanine aminotransferase. ^i^ FIB-4 liver fibrosis scores, further described in Materials and Methods. ^j^ nmol/mmol creatinine. ^k^ U-vanillylmandelic acid, µmol/mmol creatinine. ^l^ Markers of dental health: Teeth, number of teeth; ST, sound teeth, DT, decayed teeth, MT, missing teeth; DMFT, sum of DT, MT, and FT.

**Table 6:** Correlations between periodontitis markers versus AIP disease activity markers, kidney function, liver function, and catecholamine parameters.

	CAL ^i^ ≥4		PPD ^i^ ≥4		PPD ^i^ >5		BOP ^i^ %		Apical ^j^ Perio. OPG	
	Ctrl.	Case	Ctrl.	Case	Ctrl.	Case	Ctrl.	Case	Ctrl.	Case
Sodium, mmol/L	0.01	−0.13	0.04	0.01	0.08	0.11	−0.12	0.16	0.21	0.08
Potassium, mmol/L	0.23	0.14	0.04	−0.03	−0.45 **	−0.07	0.29	−0.03	0.04	−0.15
Calcium, mmol/L	−0.08	−0.02	−0.11	−0.05	0.02	−0.06	0.07	0.06	−0.20	0.00
PTH ^a^, pmol/L	−0.01	0.11	0.05	−0.09	0.14	−0.03	−0.07	−0.04	0.32 *	0.11
eGFR Creatinine ^b^	−0.47 **	−0.34 *	−0.04	−0.13	−0.09	−0.29 *	0.27	−0.07	−0.33 *	−0.09
Urate, μmol/L	0.15	0.11	0.17	0.13	0.01	0.24	0.13	−0.16	0.02	0.35 *
Carbamide, mmol/L	0.45 **	0.27	0.27	0.10	0.02	0.25	−0.07	0.13	0.05	0.01
Phosphate, mmol/L	−0.11	0.04	−0.11	0.04	−0.04	0.15	−0.31 *	0.18	−0.31 *	−0.39 **
Creatinine, µmol/L	0.33 *	0.18	−0.05	0.14	−0.04	0.28	−0.04	0.04	0.03	0.13
Albumin, g/L	−0.05	−0.24	−0.11	−0.11	0.005	−0.16	0.08	−0.41 **	−0.31 *	−0.19
U-Albumin/creat. ^c^, mg/mmol	0.16	0.12	0.17	0.06	0.11	0.04	0.15	−0.01	−0.31	0.13
S-AAT ^d^, g/L	0.08	0.21	0.33 *	0.02	0.16	0.17	0.01	0.20	−0.12	−0.05
S-Bilirubin, µmol/L	0.02	0.06	−0.13	−0.12	−0.09	0.00	0.13	0.06	−0.03	−0.12
Gamma GT ^e^, U/L	0.32 *	0.06	0.32 *	0.06	0.19	0.33 *	0.27	0.04	0.11	0.42 **
ALP ^f^, U/L	0.03	0.13	0.12	0.15	0.12	0.12	0.08	0.25	0.16	0.23
Prealbumin, g/L	0.06	−0.13	−0.25	−0.06	0.15	−0.11	−0.22	−0.16	−0.26	0.13
AST, IU/L	0.03	−0.06	−0.15	−0.18	0.04	−0.09	−0.19	−0.07	0.16	−0.18
ALT, IU/L	−0.07	−0.15	−0.13	−0.08	0.11	−0.04	0.01	−0.11	0.03	0.11
FIB4 ^g^	0.39 **	0.40 **	0.20	−0.01	0.12	0.21	−0.13	0.11	0.47 ***	−0.02
U-Epinephrine, nmol/mmol	−0.02	−0.27	−0.02	−0.07	−0.09	−0.01	0.13	−0.16	0.10	−0.03
U-Norepinephrine, nmol/mmol	0.30 *	0.25	0.27	0.07	0.08	0.22	−0.03	0.13	−0.09	0.08
U-VMA ^h^, µmol/mmol	0.13	0.23	0.05	0.35 *	0.02	0.32 *	−0.17	0.19	0.02	0.10
IgG, g/L	0.07	0.04	0.14	0.00	−0.20	−0.24	0.03	0.04	0.11	−0.19
IgA, g/L	0.12	0.06	0.21	0.13	−0.09	0.17	0.18	0.25	−0.05	0.31 *
IgM, g/L	−0.02	−0.07	−0.32 *	0.02	−0.17	0.04	−0.05	−0.05	−0.45 **	−0.03

The data represent the Spearman correlation coefficient r analyzed using the non-parametric Spearman correlation. Significant correlations are indicated in bold. The dental variables arise from 47 acute intermittent porphyria (AIP) cases and 47 matched controls. For CAL measurements n = 45 because two AIP cases had crowns covering their natural teeth, which made it impossible to measure CAL. The *p* values are exact, two-tailed. * *p* < 0.05, ** *p* < 0.01, *** *p* < 0.001. ^a^ P-parathyroid hormone. ^b^ eGFR creatinine = relative estimated glomerular filtration rate (eGFR) calculated using the Chronic Kidney Disease Epidemiology Collaboration (CKD-EPI) with creatinine equation (mL/min/1.73 m^2^). ^c^ U-albumin/creatinine. ^d^ S-alpha-1-antitrypsin. ^e^ S-gamma-glutamyl transferase. ^f^ S-alkaline phosphatase. ^g^ Liver fibrosis index 4 (FIB4) calculated as follows: (age × AST)/(platelets × (sqr. (ALT)), with age in years, aspartate aminotransferase (AST) and alanine aminotransferase (ALT) as U/L, and platelet count as 10^9^/L. ^h^ U-vanillylmandelic acid. ^i^ Markers of marginal periodontitis; clinical attachment loss (CAL), probing pocket depth (PPD), and bleeding on probing (BOP%). ^j^ Apical periodontitis as found at the orthopantogram.

**Table 7:** Inflammatory markers, porphyrin precursors, and demographic characteristics in AIP **^a^** cases with and without periodontitis as compared to controls.

	Ctrl (n = 37)	Case (n = 33)	Ctrl − PD ^b^ vs. Case − PD ^b^	Ctrl (n = 10)	Case (n = 14)	Ctrl + PD vs. Case + PD	Case − PD vs. Case + PD	Ctrl − PD vs. Ctrl + PD
Periodontitis	−	−	*p*	+	+	*p*	*p*	*p*
Age	47 (29–61)	47 (28–59)	0.99	60 (52–71)	57 (46–71)	0.59	0.048	0.02
Sympt. AIP ^a^		21 (64%)			11 (79%)			
Asympt. AIP ^a^		12 (36%)			3 (21%)			
Female, n(%)	14 (38%)	15 (45%)		6 (60%)	5 (36%)			
Male, n (%)	23 (62%)	18 (55%)		4 (40%)	9 (64%)			
Difficulties chewing	1 (3%)	0 (0%)		1 (10%)	2 (14%)			
Body mass index	26 (24–30)	26 (25–29)	0.99	30 (26–33)	29 (27–32)	0.83	0.06	0.06
Smoking pack-years	0.1 (0.0–3.5)	1.8 (0.0–9.1)	0.31	5.6 (1.1–18.1)	4.9 (0.0–30)	0.98	0.28	**0.02**
Waist/Hip ratio	1.0 (0.9–1.0)	0.9 (1.0–1–0)	0.90	1.0 (0.9–1.0)	1.0 (0.9–1.0)	0.72	0.30	0.41
Waist ^c^, cm	94 (88–103)	93 (88–103)	0.88	103 (95–110)	103 (94–113)	0.93	**0.046**	**0.04**
U-PBG ^d^	0.4 (0.3–0.5)	2.6 (0.9–10.2)	**<0.001**	0.7 (0.4–0.8)	1.4 (0.7–4.0)	**0.004**	0.35	**0.02**
U-ALA^d^	1.9 (1.6–2.3)	4.1 (2.7–7.8)	**<0.001**	1.8 (1.6–2.7)	2.9 (1.8–7.4)	0.054	0.20	0.83
U-Total porphyrins ^e^	7.1 (4.5–10.6)	28 (8.2–109)	**<0.001**	4.6 (2.7–7.0)	13 (10–43)	**0.002**	0.41	0.051
eGFR ^f^	91 (76–103)	86 (67–108)	0.79	76 (62–88)	80 (41–93)	0.91	0.21	**0.03**
PAI-1, pg/mL	4743 (3120–7205)	5136 (3218–6928)	0.82	7980 (6633–8862)	7508 (5351–9228)	0.89	**0.01**	**0.02**
IL-1β, pg/mL	0.9 (0.7–1.4)	1.6 (0.9–3.5)	**0.002**	0.8 (0.7–1.0)	1.6 (0.9–3.3)	**0.006**	1.00	0.21
IL-1RA, pg/mL	24 (13–58)	85 (14–211)	**0.007**	32 (19–40)	57 (25–161)	0.11	0.95	0.72
IL-6, pg/mL	2.0 (0.6–3.0)	5.0 (1.6–9.5)	**0.003**	2.0 (1.7–3.0)	4.5 (2.0–12)	**0.04**	0.75	0.82
IL-7, pg/mL	3.0 (1.7–5.0)	8.0 (5.0–14.5)	**<0.001**	2.5 (1.1–4.0)	6.0 (3.8–12.0)	**0.002**	0.41	0.17
CXCL8, pg/mL	6.0 (3.0–7.5)	8.0 (5.5–15)	**0.006**	4.0 (3.8–5.0)	7.5 (5.8–14)	**0.001**	0.86	0.34
IL-13, pg/mL	2.1 (1.6–4.0)	4.0 (2.0–7.0)	**0.001**	3.0 (1.6–4.5)	6.0 (3.0–12)	**0.01**	0.09	0.71
CCL11, pg/mL	34 (18–60)	84 (59–114)	**<0.001**	65 (29–77)	76 (52–99)	0.16	0.71	0.08
CXCL10, pg/mL	502 (378–785)	970 (799–1201)	**<0.001**	806 (531–1203)	763 (532–973)	0.89	0.08	0.12
VEGF, pg/mL	2.0 (0.4–5.0)	16 (5.5–30)	**<0.001**	1.8 (1.7–4.3)	16 (6.8–33)	**<0.001**	0.86	>0.99
TCC, CAU/mL	0.4 (0.3–0.5)	0.4 (0.3–0.5)	0.52	0.5 (0.4–0.6)	0.4 (0.3–0.5)	**0.04**	0.89	**0.02**
C3bc/C3 ^g^	5.3 (4.5–7.0)	7.3 (5.8–8.2)	**0.002**	5.9 (4.4–7.5)	6.1 (5.3–7.4)	0.63	0.17	0.60
C3, g/L	1.1 (1.0–1.2)	1.1 (1.0–1.2)	0.94	1.3 (1.0–1.4)	1.3 (1.1–1.3)	0.85	0.06	0.09

The data represent the median values and IQR from 47 AIP cases (Case) and 47 matched controls (Ctrl). The Mann–Whitney U-test was used. *p* values are exact, two-tailed p. Significant *p* values are indicated in bold. ^a^ Acute intermittent porphyria. ^b^ Periodontitis. ^c^ Waist circumference, cm. ^d^ μmol/mmol creatinine. ^e^ nmol/mmol creatinine. ^f^ CKD EPI eGFR Cystatin C, mL/min/1.73 m2. ^g^ Complement arbitrary units (CAU)/mL/g/L.

## Data Availability

Data supporting reported results can be obtained upon request.

## Supplementary Materials

The following supporting information can be downloaded at: https://www.mdpi.com/article/10.3390/life12081270/s1, Table S1: STROBE Statement—checklist.

## Appendix A

**Table A1:** Findings in AIP ^a^ cases on orthopantograms.

**AIP ^a^ Cases**	**Findings on Orthopantogram**
Symptomatic AIP:	
AIP 11	Operated jaw fracture with osteosynthesis plates
AIP 20	Idiopathic osteosclerosis right corpus mandibula
AIP 21	Right jaw joint: flatted caput, anterior osteophyte. Basal mandibula: idiopathic osteosclerosis
AIP 29	Agenesia or little developed maxillary sinuses, atypical trabecular pattern in mandible and maxillary bone
AIP 45	Sinus polyp in right maxillary sinus
AIP 64	Osteosclerosis, corpus mandibula
AIP 69	Bad crown works, probably gingivitis
AIP 78	Idiopathic resorption 2–8
AIP 81	A bit flattening of teeth, osteophyte anterior; obliteration pulp canal all teeth
Asymptomatic AIP:	
AIP 2	Bi-fid condyles, obliteration pulp canal all teeth
AIP4	Sinus polyp in right maxillary sinus

The data represent the comments made by the oral surgeon to the orthopantograms (OPG) from 47 acute intermittent porphyria (AIP) cases. Among the symptomatic AIP cases, 9 of the 32 got comments on the OPG. Among the asymptomatic AIP cases, 2 of the 15 got comments on OPG. In addition to this, there were found no current jawbone infections and jawbone traumas on OPG in AIP cases or controls, but one odontoma was found in a control. The number of decayed teeth, filled teeth, root canal fillings, abnormal tooth positions, crowns, bridge pillars, inflamed root canal fillings, root rests, and implants were also examined on the OPG, and when comparing them in AIP cases vs. controls we found all (*p* > 0.05), not shown. The controls also got some comments, not summarized here. ^a^ Acute intermittent porphyria, AIP.

**Table A2:** Correlations between selected cytokines and beta cell function (%B) in persons with Acute intermittent porphyria.

	**% Beta Cell Function**	
	**r-Value**	***p-*** **Value**
IL-5 ^a^	−0.31	**0.03**
IL-7 ^a^	−0.34	**0.02**
IL-10 ^a^	−0.28	**0.049**
IL-12 ^a^	−0.31	**0.03**
IL-15 ^a^	−0.33	**0.03**
CCL4 ^a^	−0.36	**0.01**
CCL11 ^a^	−0.31	**0.04**
GM-CSF ^a^	−0.35	**0.02**
TNF ^a^	−0.31	**0.03**

The data represent the Spearman correlation coefficient r analyzed using the non-parametric Spearman correlation. The variables arise from 47 acute intermittent porphyria (AIP) cases. Significant correlations are indicated in bold. The *p* values are exact, two-tailed. ^a^ IL-interleukins, CCL—chemokine ligands, GM-CSF—granulocyte-macrophage colony-stimulating factor, TNF—tumor necrosis factor.
