# Supplementary material for: Dental and Periodontal Health in Acute Intermittent Porphyria
Source: Life (Basel). 2022 Aug 19;12(8):1270. doi: 10.3390/life12081270 (PMC9410213; doi:10.3390/life12081270)
Supplement: Supplementary file 1 [file life-12-01270-s001.zip › life-1813057-supplementary.pdf]

Table S1. STROBE Statement—checklist of items that should be included in reports of observational studies

	Item No.	Recommendation	Page No.	Relevant text from manuscript
**Title and abstract**	1	(*a*) Indicate the study's design with a commonly used term in the title or the abstract	1	In 47 AIP cases and 47 matched controls, X-rays and physical examination of clinical attachment loss (CAL), probing pocket depth (PPD), and decayed missing filled teeth (DMFT) were performed.
		(*b*) Provide in the abstract an informative and balanced summary of what was done and what was found	1	In 47 AIP cases and 47 matched controls, X-rays and physical examination of clinical attachment loss (CAL), probing pocket depth (PPD), and decayed missing filled teeth (DMFT) were performed. Dietary intake was evaluated through a diet logbook. Plasma cytokines, diabetogenic hormones were measured using Multiplex analysis and urine porphobilinogen, kidney and liver function by routine methods. In conclusion; dental health in AIP cases was correlated with insulin resistance, inflammatory markers and biomarkers of kidney and liver function

**Introduction**				
Background/rationale	2	Explain the scientific background and rationale for the investigation being reported	2	AIP patients might have acute attacks of abdominal pain, nausea, vomiting, and fatigue which can be triggered by a low carbohydrate diet, drugs, stress, hormonal changes, infections, and alcohol [5]. Insufficient carbohydrate intake might induce AIP attacks and a carbohydrate intake of 55–60% of total energy intake is recommended. Glucose acts by repressing heme synthesis in the liver via insulin and peroxisome proliferator-activated cofactor-1 alpha, thereby decreasing the accumulation of toxic biochemical intermediates [7]. High sugar intake prevents porphyric attacks but could change diabetogenic hormones and affect dental health. Particularly, periodontitis sometimes goes unrecognized by the patients, and chronic inflammation from periodontitis might increase AIP disease activity [8-10].

Objectives	3	State specific objectives, including any prespecified hypotheses	2	Complications of AIP such as kidney and liver disease, chronic inflammation, vomiting during attacks, and a high attack frequency could potentially worsen dental health. We also hypothesized differences in Homeostasis model assessment (HOMA) scores for insulin resistance in AIP cases vs. controls and in those with periodontitis. Our aim was to systematically study dental health in AIP. Further, our aim was to examine if poor dental health and kidney failure might worsen AIP as chronic inflammation and kidney failure might increase disease activity.
**Methods**				
Study design	4	Present key elements of study design early in the paper	2	A case-control study of 47 adults with AIP and 47 controls, matched for age, sex, and residence was conducted from September to November 2012 in Bodø, Nordland County, Norway.
Setting	5	Describe the setting, locations, and relevant dates, including periods of recruitment, exposure, follow-up, and data collection	2	A case-control study of 47 adults with AIP and 47 controls, matched for age, sex, and residence was conducted from

				September to November 2012 in Bodø, Nordland County, Norway.
Participants	6	(*a*) *Case-control study*—Give the eligibility criteria, and the sources and methods of case ascertainment and control selection. Give the rationale for the choice of cases and controls	2,3	A case-control study of 47 adults with AIP and 47 controls, matched for age, sex, and residence. As a practically possible approach for full dental examination of the rare disease AIP we included 50 AIP cases and 50 healthy controls. Of the AIP cases, 15 were asymptomatic and 32 symptomatic. Of the initial 50 AIP cases and 50 controls, three AIP cases and one control were edentulous. These and their matched controls were excluded [8]. Another two AIP cases and their corresponding controls were excluded only from CAL measurements because the two AIP cases had crowns covering their natural teeth, which made it impossible to measure CAL.
		(*b*) *Case-control study*—For matched studies, give matching criteria and the number of controls per case	2	A case-control study of 47 adults with AIP and 47 controls, matched for age, sex, and residence. As a practically possible approach for full dental examination of the rare disease

				AIP we included 50 AIP cases and 50 healthy controls. Of the AIP cases, 15 were asymptomatic and 32 symptomatic.
Variables	7	Clearly define all outcomes, exposures, predictors, potential confounders, and effect modifiers. Give diagnostic criteria, if applicable	3,4,5	Periodontitis was assessed by probe UNC15 with defined spring force 0.2 N. Probing pocket depth (PPD) and clinical attachment loss (CAL) were measured at four sites. As a practically feasible procedure, probing pocket depth (PPD) and clinical attachment loss (CAL) were measured at four sites, not 6 sites, due to available time and resources. Our chosen definition of periodontitis was three or more PPDs of 5 mm or more on different teeth, third molars excluded [18,19]. The number of sites with PPD≥4 mm and PPD>5 mm, and the number of teeth with CAL≥4 mm was recorded. Bleeding on probing (BOP) was measured and BOP% was calculated as the number of bleeding sites (n)/sites measured (n) x 100.
Data sources/ measurement	8*	For each variable of interest, give sources of data and details of methods of assessment (measurement). Describe comparability of assessment methods if there is more than one group	3,4,5	Twenty-seven cytokines in EDTA plasma (P) were analyzed as described in a previ-

				ous study using Multiplex Technology in a case-control study with 50 AIP cases. Furthermore, analysis of P-Insulin, P-C-peptide, P-Gastric inhibitory polypeptide, P-Visfatin, P-Resistin, P-Leptin, P-Ghrelin and P-Plasminogen activator inhibitor-1, all given in pg/mL were performed using the Bio-Plex 200 system (Bio-Rad) and a Bioplex Pro human diabetes immunoassay kit.
Bias	9	Describe any efforts to address potential sources of bias	3	One dentist evaluated all the bitewing x-rays in conjunction with the clinical examination. To minimize differences in examination technique, the dentist had, prior to the study, tested the research examination procedure. The procedure was done on individuals not part of the study, to approximate time use and to rehearse a standardized procedure.
Study size	10	Explain how the study size was arrived at	3	We had no data, prior to the study, regarding the expected mean difference on dental health between the healthy control group and a group of persons with the rare disease AIP. We anticipated for PBG in

	urine, a difference of 9 μmol/mmol creatinine between the mean value of the healthy controls and the AIP cases (data from a previous study in Sweeden). In that regard, we needed a mini-mum of 20 persons to find a statistical difference on PBG between the groups with 95% probability (P < 0.05) at the previously observed SD for mean value. Reviewing literature regarding dental research, we found that sample size often was larger than this in order to find statistical difference. As a practically possible approach for full dental examination of the rare disease AIP we included 50 AIP cases and 50 healthy controls.

Continued on next page

Quantitative variables	11	Explain how quantitative variables were handled in the analyses. If applicable, describe which groupings were chosen and why	5	The statistical analysis was performed using Prism version 6.0 from GraphPad Software Inc. (CA, USA).<br>An excel spreadsheet from the University of Oxford was used to estimate beta cell function, HOMA%B (%B), insulin sensitivity, HOMA%S (%S) and insulin resistance HOMA-IR (IR), based on serum glucose and plasma C-peptide
Statistical methods	12	(*a*) Describe all statistical methods, including those used to control for confounding	5	The Wilcoxon matched-pairs signed-rank test was used on the matched case-control data. While the Spearman nonparametric correlation was used on case and control data, the Mann-Whitney U-test was used on non-matched data. Fischer's exact test was per-formed for categorical variables. $P < 0.05$ was considered statistically significant. The statistical analysis was performed using Prism version 6.0 from GraphPad Software Inc. (CA, USA).
		(*b*) Describe any methods used to examine subgroups and interactions	5-12, tables 1-7	See tables 1-7 (including description)
		(*c*) Explain how missing data were addressed	2,3, table 1-2	Of the initial 50 AIP cases and 50 controls, three AIP cases and one control were edentulous. These and

				their matched controls were excluded [8]. Another two AIP cases and their corresponding controls were excluded only from CAL measurements because the two AIP cases had crowns covering their natural teeth, which made it impossible to measure CAL. The diet data is from 43 matched pairs
		Case-control study—If applicable, explain how matching of cases and controls was addressed	2,3	Of the initial 50 AIP cases and 50 controls, three AIP cases and one control were edentulous. These and their matched controls were excluded [8]. Another two AIP cases and their corresponding controls were excluded only from CAL measurements because the two AIP cases had crowns covering their natural teeth, which made it impossible to measure CAL.
		(*e*) Describe any sensitivity analyses		
**Results**				
Participants	13*	(a) Report numbers of individuals at each stage of study—eg numbers potentially eligible, examined for eligibility, confirmed eligible, included in the study, completing follow-up, and analysed	2-3, 5-12, tables 1-7	Of the initial 50 AIP cases and 50 controls, three AIP cases and one control were edentulous. These and their matched controls were excluded [8]. Another two AIP cases and their corresponding controls were excluded only from CAL measurements because the

				two AIP cases had crowns covering their natural teeth, which made it impossible to measure CAL
		(b) Give reasons for non-participation at each stage	5-12, tables 1-7	Of the initial 50 AIP cases and 50 controls, three AIP cases and one control were edentulous. These and their matched controls were excluded [8]. Another two AIP cases and their corresponding controls were excluded only from CAL measurements because the two AIP cases had crowns covering their natural teeth, which made it impossible to measure CAL.
		(c) Consider use of a flow diagram		Not performed
Descriptive data	14*	(a) Give characteristics of study participants (eg demographic, clinical, social) and information on exposures and potential confounders	5	The baseline demographic characteristics of the AIP population and the controls were equal on most variables (Table 1).
		(b) Indicate number of participants with missing data for each variable of interest	2,3 and description in tables 1-7	A case-control study of 47 adults with AIP and 47 controls Three AIP cases and one control were edentulous. These and their matched controls were excluded [8]. Another two AIP cases and their corresponding controls were excluded only from CAL measurements because the two AIP cases had crowns covering their natural teeth, which made it impossible to measure CAL.

				The diet data is from 43 matched pairs.
Outcome data	15*	*Case-control study*—Report numbers in each exposure category, or summary measures of exposure	5-12, Tables 1-7	Periodontitis (+/-PD):<br>Ctrl -PD n=33, Case -PD n=30, Ctrl +PD n=10 and Case +PD n=13. (Table 2).
Main results	16	(*a*) Give unadjusted estimates and, if applicable, confounder-adjusted estimates and their precision (eg, 95% confidence interval). Make clear which confounders were adjusted for and why they were included	5-12, tables 1-7	See tables 1-7 (including description)
		(*b*) Report category boundaries when continuous variables were categorized	5-12, tables 1-7	See tables 1-7 (including description)
		(*c*) If relevant, consider translating estimates of relative risk into absolute risk for a meaningful time period		

Continued on next page

Other analyses	17	Report other analyses done—eg analyses of subgroups and interactions, and sensitivity analyses	Appendix A	The data represents the comments made by the oral surgeon to the orthopantograms (OPG) from 47 acute intermittent porphyria (AIP) cases.
**Discussion**				
Key results	18	Summarise key results with reference to study objectives	16	The carbohydrate intake was significantly higher in AIP cases with periodontitis compared to controls with periodontitis. Simultaneously, insulin and C-peptide levels were significantly higher in the AIP cases with periodontitis versus AIP cases without. Periodontitis in AIP was also associated with higher insulin resistance. In cases with AIP, lower beta cell function was associated with inflammation. Decreased renal function in AIP was associated with increased number of MT and increased PTH. In the AIP cases, liver and kidney markers, smoking status, and inflammatory markers were correlated with reduced dental health.
Limitations	19	Discuss limitations of the study, taking into account sources of potential bias or imprecision. Discuss both direction and magnitude of any potential bias	15	A limitation is that dental hygiene habits, including the use of fluoride and snuff, was not recorded and previous dental records were not available. There is also a relatively

				small number of participants in this study, and there is a relatively large number of statistical tests performed. This could have led to false positive results. Our lack of power calculations regarding dental health in AIP, since there were no data on expected dental health in AIP in the literature, in combination with the rarity of the disease, may have led to low power which may have reduced the chance of detecting a true effect, and it might also have reduced the likelihood that our statistically significant re-sults were actually reflecting a true effect.
Interpretation	20	Give a cautious overall interpretation of results considering objectives, limitations, multiplicity of analyses, results from similar studies, and other relevant evidence	16	These results suggest that AIP-associated organ complications and chronic low-grade inflammation may worsen dental health, although due to the small sample size, the possibility of false positive findings cannot be excluded. Bearing in mind that the results from small subgroups could be less reliable, we anyhow cautiously suggest a hypothesis that inflammation induced by poor dental health in some cases might in turn worsen AIP.
Generalisability	21	Discuss the generalisability (external validity) of the study results		Most participants

				had the same AIP mutation and were from Norway. This may affect the external validity, but on the other hand the same mutation is also prevalent in Sweden, and all the AIP mutations lead to reduced function of the same enzyme.
**Other information**				
Funding	22	Give the source of funding and the role of the funders for the present study and, if applicable, for the original study on which the present article is based	16	Funding: This research was supported by grants from the Somatic Research Fund at Nordland Hospital Trust, Northern, Southern, and Eastern Norway Regional Health Authorities, the Re-search Council of Norway, and the European Community's Seventh Framework Program under grant agreement n° 602699 (DIREKT) and the Odd Fellow Foundation.

*Give information separately for cases and controls in case-control studies and, if applicable, for exposed and unexposed groups in cohort and cross-sectional studies.

Note: An Explanation and Elaboration article discusses each checklist item and gives methodological background and published examples of transparent reporting. The STROBE checklist is best used in conjunction with this article (freely available on the Web sites of PLoS Medicine at http://www.plosmedicine.org/, Annals of Internal Medicine at http://www.annals.org/, and Epidemiology at http://www.epidem.com/). Information on the STROBE Initiative is available at www.strobe-statement.org.
